# Transition to annual life history coincides with reduction in cell cycle speed during early cleavage in three independent clades of annual killifish

**DOI:** 10.1186/2041-9139-5-32

**Published:** 2014-09-22

**Authors:** Luca Dolfi, Roberto Ripa, Alessandro Cellerino

**Affiliations:** 1 Scuola Normale Superiore, Pisa, Italy; 2 Fritz Lipmann Institute for Age Research, Leibniz Institute, Leibniz, Germany

**Keywords:** Nothobranchius, Early development, Segmentation, Life history evolution, Midblastula transition, Imaging, Diapause

## Abstract

**Background:**

Annual killifishes inhabit temporary ponds and their embryos survive the dry season encased in the mud by entering diapause, a process that arrests embryonic development during hostile conditions. Annual killifishes are present within three clades distributed in Africa (one East and one West of the Dahomey gap) and South America. Within each of these phylogenetic clades, a non-annual clade is sister *taxon* to a annual clade and therefore represent an example of convergent evolution.

Early cleavage of teleost embryos is characterized by a very fast cell cycle (15-30 minutes) and lack of G_1_ and G_2_ phases. Here, we decided to investigate rates of early cleavage in annual killifishes. In addition, we specifically tested whether also annual killifish embryos lack G_1_ and G_2_ phases.

**Results:**

We used time lapse brightfield microscopy to investigate cell division kinetics during the first developmental stages of annual- and non-annual species belonging to the three different phylogenetic clades. Annual killifishes of all three clades showed cleavage times significantly longer when compared to their non-annual sister taxa (average 35 min vs. average 75 min). Using FUCCI fluorescent imaging of the cell cycle after microinjection in the annual species *Nothobranchius furzeri*, we demonstrate that the first 5 division are synchronous and do not show a G_1_ phase. Cell cycle synchronization is lost after the 5th cleavage division.

**Conclusions:**

Our results show, for the first time, that cell cycle rate during cleavage, a trait thought to be rather evolutionary conserved can undergo convergent evolutionary change in response to variations in life-history.

## Background

Life history trade-offs are major forces that shape the evolution of embryonic development. Extreme examples are seasonal species where embryos undergo *diapause*, a suspension of development to overcome hostile periods. A seasonal life cycle has evolved also in a clade of teleost fishes (suborder Aplocheiloidei) known as *annual* killifish [1]. Annual killifish inhabit ephemeral bodies of water that fill during the monsoon season and disappear by evaporation after its end. Annual killifish are present in Africa and South America and are adapted to alternating wet and dry seasons. All adult fish die when their habitat dries out and survival of the population is ensured by desiccation-resistant eggs that enter into diapause and remain encased in the dry mud until the next rainy season [2-5]. Annual killifish, especially those of the genus *Nothobranchius*, are becoming popular model organisms for studying aging and life-history evolution [3,6-16]. Diapause in annual killifish is associated with major metabolic remodeling, where several pathways involved in energy production are modulated in order to minimize the embryo's aerobic metabolism and production of reactive oxygen species allowing quiescence. During diapause, oxygen consumption is suppressed and the cell cycle arrested [4,17]. In some habitats of annual killifish the duration of the temporary pools is only a few months [15], and therefore the animals spend the majority of their life in diapause.

The morphogenetic processes of early embryonic development of annual killifish show a remarkable derived character: during epiboly, blastomeres migrate as dispersed cells and remain on the yolk surface for days before they finally re-aggregate to form the embryonic axis [2]. This unique stage of development is often associated with the ability to enter into stasis at diapause I, although this trait varies among species of annual killifish.

During this dispersed state, the blastomeres migrate randomly for days before starting the reaggregation process [18] (see Additional file 1). After formation of the embryonic axis, the development is arrested at a specific stage of somitogenesis in diapause II [2] where the embryo can remain for several months (or even years).

**Figure 1:**
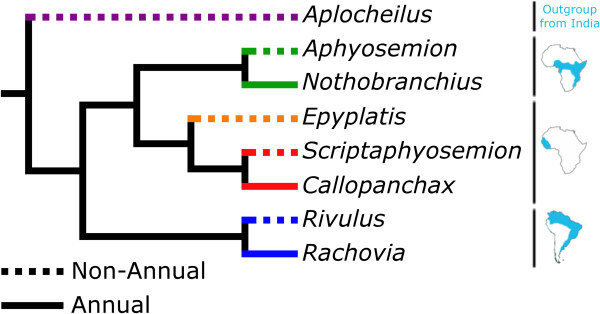
**Phylogram of the species used in the present study.** Dashed lines indicate non-annual species, solid lines annual species and color codes for the three evolutionary lineages. The geographic distribution of each lineage is show on the right. The phylogram is derived from the original Murphy and Collier molecular phylogram [19] based on cytochrome b, 12 s rRNA and 16 s rRNA genes.

Annual killifish are divided into three clades distributed in Africa (one west and one east of the Dahomey gap) and in South America (Figure 1) according to the only comprehenvise molecular phylogeny for this taxon based on mitochondrial *loci*. In each of these regions, one clade is found that contains both annual and non-annual *genera* that are sister *taxa*[19,20]. The outgroup of all annual killifish is the non-annual genus Aplocheilus. Murphy and Collier [19] interpret his distribution of annual and non-annual clades with a model whereby the most common ancestor of all annual killifish was annual, and non-annual clades arose due to three independent losses of the ancestral annual trait [19,20]. Annual killifish offer a unique paradigm for investigations of the phenotypic evolution correlated with transitions in life history. As the three annual clades evolved independently, any trait that is common to these clades is likely to be the result of evolutionary convergence in response to transition to annual life history.

Here we investigated the early development (up to the sixth division, cleavage) of annual and non-annual killifish embryos. In the species most closely related to those we study here, *Fundulus heteroclitus*[21], it is well described that during the cleavage phase cell divisions are synchronized so that 2, 4, 8, 16 and 32 cells arise in succession. This pattern is broadly conserved in teleost species, and cell cycle during cleavage is extremely fast (in the order of 15 to 30 minutes) in typical model teleosts such as *Danio rerio*[22], *Oryzia latipes*[23], *Gasterosteus aculeatus*[24] and also* Xenopus laevis*[25]. During cleavage there is no transcription of the embryonic genome but only translation of maternal transcripts and the cell cycle lacks the G_1_ and G_2_ phases, thus proceeding directly from S- to M-phase [26,27]. Here, we investigated the rate of early cleavages in annual killifishes embryos to assess whether cell-cycle length evolved differently in annual and non-annual clades.

## Methods

### Fish maintenance

All the fish used were raised in 35-L tanks at 23 to 25°C. They were fed two to three times a day with frozen Chironomus larvae or living nauplii of *Artemia salina*, depending on size. Annual killifish were bred in a small 3-L tank with 2 cm of river sand on the bottom for 1 hour. Eggs were collected by sieving the sand with a plastic net. Non-annual killifish were bred by floating an artificial floating bush made of acrylic wool strings (spawning mop) in the tank for 1 hour. Eggs were collected from the mop by hand.

### Bright-field imaging

After collection, embryos were sorted and dead eggs and those past the two-cell stage were removed. Chorion projections were removed from three to six of the remaining embryos with forceps (when possible) and embedded in 1.5% SeaPlaque low gelling temperature agarose in a Petri dish. While the agar was still molten, eggs were put on the bottom of the dish and oriented, so that the developing blastodisc (animal pole) was parallel to the dish floor. Time lapse images were captured using a Leica M80 stereomicroscope placed in a temperature-controlled room held at 26°C. Magnification ranged between 25 and 45 times, depending on species. Photos were acquired every two to five minutes, depending on species, with a Nikon Digital Sight DS-Fi1 camera using Nikon Elements F 3.0 software or with a Zeiss AxioCam ERc5s camera using ZEN software. Photos from a single acquisition session were opened with Fiji ImageJ as an image sequence, synchrony between eggs of the same experiment was evaluated and timings at which the different cell divisions occurred were noted.

### FUCCI imaging

FUCCI reporter constructs used were previously optimized in for zebrafish [27]. Geminin-azami green and cdt1-kusabira orange were extracted from plasmids pT2KXIG using BamHI and ClaI and sub-cloned in a PcS2 expression vector under the control of an SP6 promoter. Synthetic RNAs were transcibed using mMESSAGE mMACHINE® SP6 Transcription Kit, Ambion: 1 nl of both RNAs (final concentration 400 pg/nl each) was injected intracellularly in one-cell-stage embryos using a Tritech microINJECTOR™. Injected embryos were then embedded in 2% low melting agar in a wilkon dish, oriented and left for 1 hour at 25°C while the agar was hardening.

Fluorescence images were acquired using a Leica TCS SP2 confocal microscope; 250 to 350 μm were scanned and an image was taken every 6 μm (40 to 60 images per embryo in total) for each time point. Acquisitions were performed every 13 minutes for almost 24 hours at room temperature (RT, using 488 and 543 emission lasers. Single images were reconstructed from stacks using Fiji imageJ. All images were edited with GIMP. Colors, contrast, brightness and sharpness were adjusted in order to optimize the contrast of the images. All videos were edited with Sony Vegas™. A sequence of images from a single embryo was chosen, oriented, cropped and saved as a new image sequence. This sequence of images was imported as a continuous video in Sony Vegas™.

## Results and discussion

**Figure 2:**
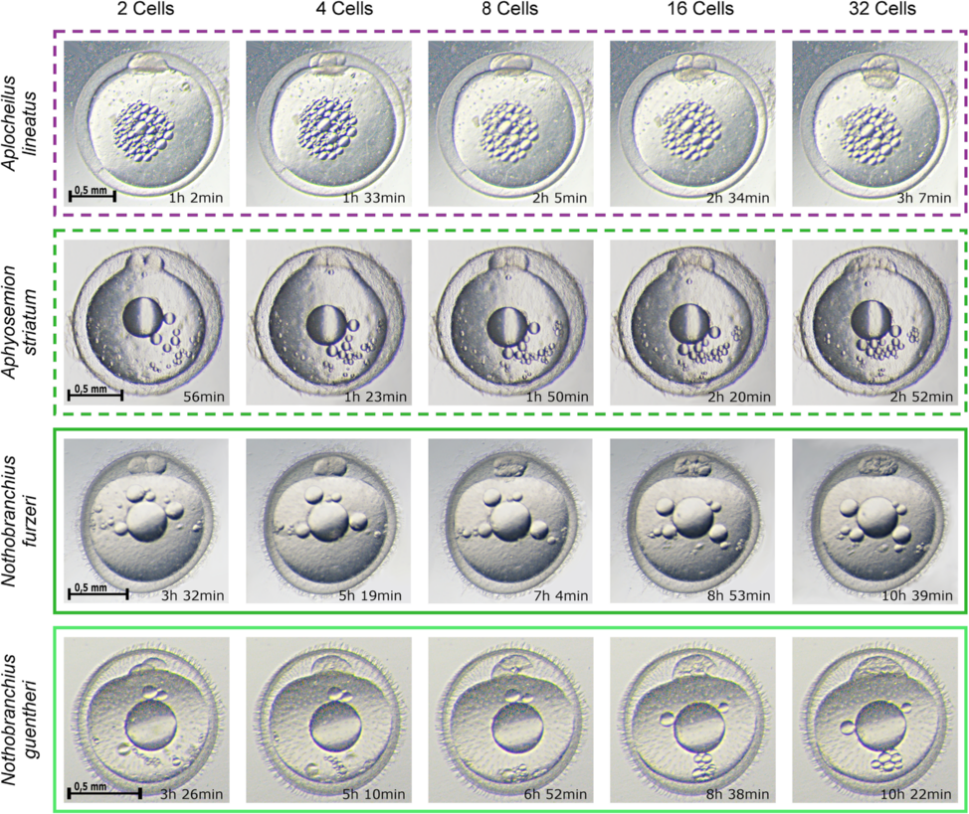
**Early cleavage time-lapse.** Non-annual species (dashed boxes) are compared with annual species (solid boxes) by brightfield time lapse imaging. Early cleavage stages are shown for each species and the average time at which they occur is indicated. There is a large difference in early cell division rates between annual and non-annual species.

**Figure 3:**
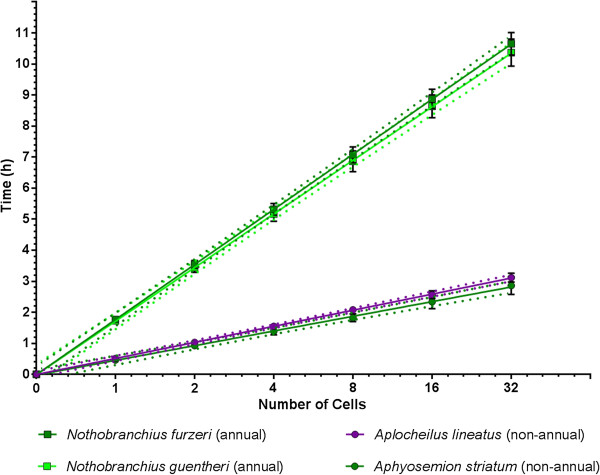
**Early division rates greatly differ between annual and non-annual species.** Time-lapses videos were plotted, with developmental stages on the x-axis and time of occurrence on y-axis. Data are means of three independent experiments. Error bars represent standard deviations. Dashed lines indicates confidence intervals of the regressions. The slopes of the lines clearly show the great difference between annual and non-annual early division times.

**Figure 4:**
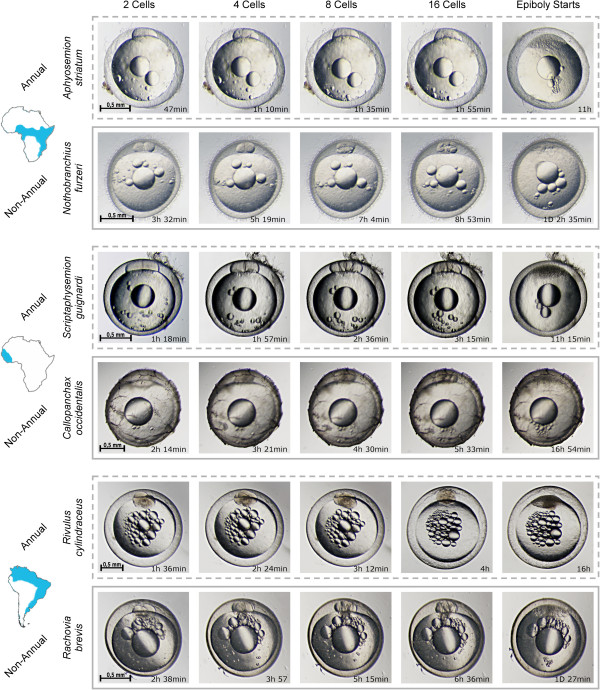
**Early cleavage time-lapse.** Non-annual species (dashed boxes) are compared with annual species (solid boxes) by brightfield time-lapse imaging. Early cleavage stages are shown for each species and the time at which they occur is indicated. There is a large difference in early division rate between annual and non-annual species.

We used time-lapse microscopy to follow the early cleavage phase of the annual species *Nothobranchius furzeri* and *N. guentheri*, their closest non-annual relative *Aphyosemion striatum,* and the closest outgroup *taxon* to all annual killifish *Aplocheilus lineatus* (Figure 2 and Additional file 2). The length of the cell cycle varied dramatically between these species. The average doubling time was 106,5 minutes +/-2,2, 103,6 minutes +/- 2,6, 28,2 minutes +/- 1,5 and 31,1 +/- 0,8 for *N. furzeri (annual)*, *N. guentheri (annual)*, *A. striatum (non-annual)* and *A. lineatus (non-annual)*, respectively. Slopes of the regression lines between each pair of annual and non-annual species were different at *P* <0.0001 (Figure 3). As a consequence, *A. striatum* embryos contain 32 cells after 3 hours while at the same time *N. furzeri* has just completed the first cleavage. To test whether this striking difference between annual and non-annual sister clades was observed in all evolutionary lineages, we followed the early cleavage phase of 11 different species of Aplocheiloidei representing the three different evolutionary lineages. From South America, we imaged the annual species *Rachovia brevis* and the related non-annual species *Rivulus cylindraceus*. From Africa, west of Dahomey gap, we imaged the annual species *Callopanchax occidentalis* and the non-annual species *Scriptaphysemion guignardi* and *Epiplatys dageti monroviae*. From Africa, east of Dahomey gap, we imaged the annual species *Nothobranchius furzeri*, *N. guentheri*, *N. korthause* and *N. melanospilus* and the non-annual species *Aphyosemion australe* and *A. striatum* (Figure 4).

**Figure 5:**
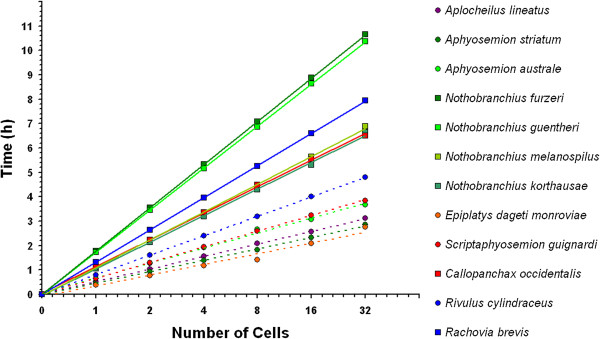
**Difference between annual and non-annual division rate is conserved in Aplocheloidei.** All species followed with time-lapses videos were plotted. Developmental stages are shown on the x-axis and time at which they occur on the y-axis. Dashed lines indicate non-annual species, solid lines annual species and color codes the geographic clade. For each species only one individual embryo is plotted.

In Figure 5, the time at which each cell division occurs is reported for each annual (solid line) and non-annual (dashed line) species, whereas color codes show the geographic origin of the species. The main result of our study is that the length of the cell cycle varies dramatically between annual and non-annual species (Figure 5, Student’s t-test, *P* <0.001) and that this difference evolved at least three times during Aplocheloid history. The six non-annual species show an average cleavage time of 34.8 minutes (range 23.0 to 48.0). This rate is comparable to the outgroup *A. lineatus*. On the other hand, the seven annual species show an average cleavage time of 75.6 minutes (range 66.0 to 100.0).

**Figure 6:**
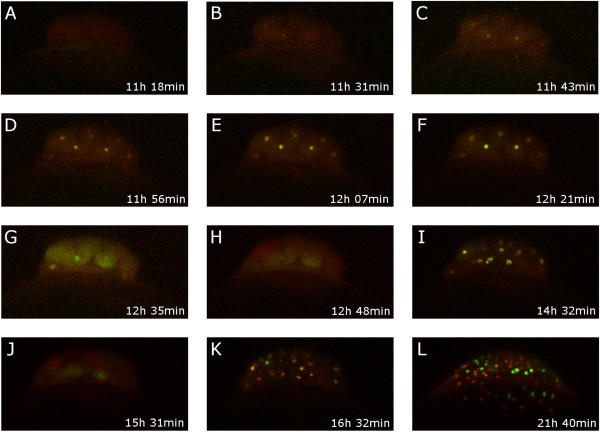
**Cell cycle during early cleavage.** Cell cycle progression in *Nothobranchius furzeri* was visualized by fluorescent ubiquitination-based cell cycle indicator (FUCCI). From **(A) **to **(H)** the last synchronus division (fourth) is shown, all nuclei are yellow and cells perfectly synchronized in cycling. **(I)** Represents the first division where asynchrony starts. **(J)** last synchronous dark stage. **(K-L)** later stages when cells are clearly asynchronous and cells in different phases can be recognized at the same time point.

There are two possible explanations for these results: a) annual fish have slower S- and M-phases, or b) annual species have inserted a G_1_ phase in the cell cycle during early cleavage. To distinguish between these two competing hypotheses, we took advantage of the fluorescent ubiquitination-based cell cycle indicator (FUCCI) system for imaging cell cycle progression. In this system, a red and a green fluorescent reporter are fused to protein motives that drive degradation in the G_1_ and the S phase respectively [28]. Therefore, a cell in the G_1_ phase would appear red and a cell in the S/G_2_ phase green, while cells in the M- phase would show no fluorescence. FUCCI reporter mRNAs optimized for zebrafish [29] were microinjected in *N. furzeri* fertilized eggs and the fluorescence followed over time (five independent experiments). From these experiments (Figure 6 and Additional file 3), it is clear that the first five divisions are synchronous. The red and green fluorescent reporters rise in phase after cell division (Figure 6A-F), become cytoplasmic (Figure 6G) and are then degraded (Figure 6H) before cell division. Starting from the sixth division (Figure 6 K), cells with a prevalence of red fluorescence intermingled with cells with a prevalence of green fluorescence are detectable, demonstrating desynchronization of the cell cycle and the appearance of a G_1_ phase.

**Figure 7:**
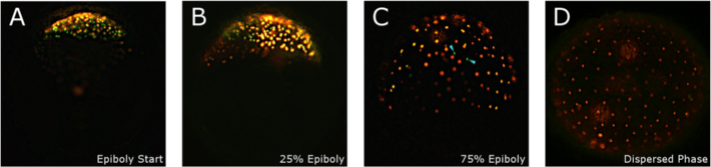
**Cell cycle during epiboly and dispersed phase.** Cell cycle progression in *Nothobranchius furzeri* was visualized by fluorescent ubiquitination-based cell cycle indicator (FUCCI). Green cells in the S-/G_2_ phase, red cells are G_1_-phase cells. Cells divide for the first days of development at the animal pole **(A-B)** and then just migrate in G1 onto the embryo surface during late epiboly and the dispersed cell phase **(C-D)**.

At the start of epiboly, cells continue dividing in the animal pole while migrating (Figure 7A-B) for almost 2 days (at 26°C). As epiboly proceeds (days 3 to 4), the fraction of cells in the S-phase is progressively reduced and at 70% epiboly only a few scattered cells appear to be in the S-phase (arrows, Figure 7C). Once epiboly is complete, the dispersed phase starts and lasts for 5 days. During the dispersed cell phase, all cells appear to be in G_1_ as indicated by the detection of red fluorescence only (Figure 7 D).

The results of the FUCCI imaging indicate that the slowness of the cell cycle in annual killifish species is not due to the presence of a G_1_ phase, but rather to a retardation of the S- and M-phases. Further, the first signs of asynchrony are observed already between the fifth and sixth divisions. This is considerably earlier than the tenth division, as originally reported for *D rerio* in correspondence to the mid blastula transition [30], and is in line with recent results obtained in *O latipes,* where the desynchronization is observed between the fifth and the sixth division [31]. In medaka and also in zebrafish, however, the first signs of transcription from the zygotic genome are observed around the sixth division [31,32] and are defined as pre-MBT transcription. It is possible that in annual fish as well, the activation of the very first zygotic genes corresponds to the first signs of asynchrony.

A striking difference in cell-cycle length between annual and non-annual species was observed in three independent clades of killifish. This datum strongly suggests that transition of life history was the driving force for this phenotypic switch. It is however unclear whether a slow cell cycle provides any evolutionary advantage. Prolongation of cell-cycle in annual killifish could result either from the loss of an evolutionary constraint or from the appearance of a novel evolutionary constraint. In the first scenario, the slow cell-cycle evolved in response to the weakening of a directional selection for rapid development that applies to non-annual killifish, because annual killifish eggs spend months (or even years) before hatching. It is indeed of interest if annualism is the basal trait of all three clades and the non-annual clades lost this basal trait independently. Therefore, in all three in stances, transition to a non-annual lifestyle induced a convergent evolution of fast cell-cycle likely because of re-establishment of a directional selection for developmental speed.

In the second scenario, cell cycle is extended as a result of a specific adaptation in order to prolong a period when the embryo can respond with phenotypic plasticity to environmental conditions. In fact, annual killifish embryos can skip diapause when environmental conditions are favorable [33]. Diapause skipping can be an advantage if the water body fills more than once during a season [34] allowing a second generation and the commitment to this alternative developmental pathway is determined during the early development [33]. So, slowing the cleavage phase would prolong the amount of time available to embryos for responding to environmental conditions.

## Conclusions

Our results show, for the first time, that cell-cycle rate during cleavage, a trait thought to be rather evolutionary conserved can undergo convergent evolutionary change in response to variations in life history.

### Ethics

The experiments did not cause any distress to the animals. The protocols of fish maintenance were authorized by the Italian Ministry of Health (Authorization number 96/2003-A).

## Abbreviations

FUCCI: Fluorescent ubiquitination-based cell cycle indicator; RT: Room temperature.

## Competing interests

The authors declare that they have no competing interests.

## Authors’ contributions

LD performed the imaging and microinjection, analyzed the data, prepared the figures/movies and wrote the manuscript. RR produced the plasmids and RNA for the study, AC designed the study, analyzed the data and wrote the manuscript. All authors read and approved the final manuscript.

## Supplementary Material

Additional file 1 ***Nothobranchius furzeri* time lapse.** A *Nothobranchius furzeri* embryo has been followed through bright-field time lapse imaging from the one-cell stage to the dispersed phase. Cells divide at the animal pole (top part, above the yolk) and then migrate over the yolk surface (sec 21) during epiboly. Macroblastomers (big cells) can be spotted moving randomly and following the epiboly migration layer. When epiboly is complete (sec 54) macroblastomers continue moving randomly during the dispersed phase, that will last for several days. Click here for file

Additional file 2 **Annual and non-annual time lapse: comparison between the division rate of annual (*Nothobranchius furzeri*) and non-annual (*Aphyosemion striatum*).** Cells divide at the animal pole (top part, above the yolk) and the time at which they cleave is greatly different between the annual and the non-annual species. Click here for file

Additional file 3 **Fluorescent ubiquitination-based cell cycle indicator (FUCCI) time lapse.** Cell cycle progression is visualized by FUCCI. Green cells are dividing cells in the S-/G_2_ phase, red cells are G_1_-phase cells; most of the fluorescent protein accumulates in the nuclei. Cells divide synchronously (appear and disappear together) until 32 cells are reached, after which asynchrony starts and cells start to cycle and divide at different times (different colors of cells at the same time, and different timing of appearing/disappearing). Click here for file

## References

[B1] Myers GS Annual fishes Aquarium J 1952 23 125 141

[B2] Wourms JP The developmental biology of annual fishes. 3. Pre-embryonic and embryonic diapause of variable duration in the eggs of annual fishes J Exp Zool 1972 182 389 414 10.1002/jez.1401820310 4674089

[B3] Genade T Benedetti M Terzibasi E Roncaglia P Valenzano DR Cattaneo A Cellerino A Annual fishes of the genus *Nothobranchius* as a model system for aging research Aging Cell 2005 4 223 233 10.1111/j.1474-9726.2005.00165.x 16164422

[B4] Podrabsky JE Hand SC The bioenergetics of embryonic diapause in an annual killifish, *austrofundulus limnaeus* J Exp Biol 1999 202 Pt 19 2567 2580 10482717 10.1242/jeb.202.19.2567

[B5] Podrabsky JE Carpenter JF Hand SC Survival of water stress in annual fish embryos: dehydration avoidance and egg envelope amyloid fibers Am J Physiol Regul Integr Comp Physiol 2001 280 123 131 10.1152/ajpregu.2001.280.1.R123 11124142

[B6] Blazek R Polacik MJ Reichard M Rapid growth, early maturation and short generation time in African annual fishes EvoDevo 2013 4 24 10.1186/2041-9139-4-24 24007640 PMC3844391

[B7] Di Cicco E Tozzini ET Rossi G Cellerino A The short-lived annual fish *Nothobranchius furzeri* shows a typical teleost aging process reinforced by high incidence of age-dependent neoplasias Exp Gerontol 2011 46 249 256 10.1016/j.exger.2010.10.011 21056099

[B8] Genade T Lang DM Resveratrol extends lifespan and preserves glia but not neurons of the *Nothobranchius guentheri* optic tectum Exp Gerontol 2013 48 202 212 10.1016/j.exger.2012.11.013 23220248

[B9] Hartmann N Reichwald K Lechel A Graf M Kirschner J Dorn A Terzibasi E Wellner J Platzer M Rudolph KL Cellerino A Englert C Telomeres shorten while Tert expression increases during ageing of the short-lived fish *Nothobranchius furzeri* Mech Ageing Dev 2009 130 290 296 10.1016/j.mad.2009.01.003 19428446

[B10] Herrera M Jagadeeswaran P Annual fish as a genetic model for aging J Gerontol A Biol Sci Med 2004 59 101 107 10.1093/gerona/59.2.B101 14999022

[B11] Hsu CY Chiu YC Ambient temperature influences aging in an annual fish (*Nothobranchius rachovii*) Aging Cell 2009 8 726 737 10.1111/j.1474-9726.2009.00525.x 19780720

[B12] Kirschner J Weber D Neuschl C Franke A Bottger M Zielke L Powalsky E Groth M Shagin D Petzold A Hartmann N Englert C Brockmann GA Platzer M Cellerino A Reichwald K Mapping of quantitative trait loci controlling lifespan in the short-lived fish *Nothobranchius furzeri*- a new vertebrate model for age research Aging Cell 2012 11 252 261 10.1111/j.1474-9726.2011.00780.x 22221414 PMC3437503

[B13] Lucas-Sanchez A Almaida-Pagan PF Madrid JA de Costa J Mendiola P Age-related changes in fatty acid profile and locomotor activity rhythms in *Nothobranchius korthausae* Exp Gerontol 2011 46 970 978 10.1016/j.exger.2011.08.009 21896325

[B14] Terzibasi E Lefrancois C Domenici P Hartmann N Graf M Cellerino A Effects of dietary restriction on mortality and age-related phenotypes in the short-lived fish *Nothobranchius furzeri* Aging Cell 2009 8 88 99 10.1111/j.1474-9726.2009.00455.x 19302373

[B15] Terzibasi Tozzini ET Dorn A Ng'oma E Polacik M Blazek R Reichwald K Petzold A Watters B Reichard M Cellerino A Parallel evolution of senescence in annual fishes in response to extrinsic mortality BMC Evol Biol 2013 13 77 10.1186/1471-2148-13-77 23551990 PMC3623659

[B16] Polacik M Blazek R Rezucha R Vrtilek M Terzibasi Tozzini E Reichard M Alternative intrapopulation life-history strategies and their trade-offs in an African annual fish J Evol Biol 2014 27 854 865 10.1111/jeb.12359 24666645

[B17] Podrabsky JE Culpepper KM Cell cycle regulation during development and dormancy in embryos of the annual killifish *Austrofundulus limnaeus* Cell Cycle 2012 11 1697 1704 10.4161/cc.19881 22531486 PMC3372388

[B18] Lesseps RJ van Kessel AH Denuce JM Cell patterns and cell movements during early development of an annual fish, *Nothobranchius neumanni* J Exp Zool 1975 193 137 146 10.1002/jez.1401930203 1176898

[B19] Murphy WJ Collier GE A molecular phylogeny for aplocheiloid fishes (Atherinomorpha, Cyprinodontiformes): the role of vicariance and the origins of annualism Mol Biol Evol 1997 14 790 799 10.1093/oxfordjournals.molbev.a025819 9254916

[B20] Hrbek T Larson A The evolution of diapause in the killifish family Rivulidae (Atherinomorpha, Cyprinodontiformes): A molecular phylogenetic and biogeographic perspective Evolution 1999 53 1200 1216 10.2307/2640823 28565541

[B21] Oppenheimer JM The normal stages of *Fundulus heteroclitus* Anat 1937 68 1 15

[B22] Kimmel CB Ballard WW Kimmel SR Ullmann B Schilling TF Stages of embryonic development of the Zebrafish Developmental Dynamics 1995 203 253 310 10.1002/aja.1002030302 8589427

[B23] Iwamatsu T Stages of normal development in the medaka *Oryzias latipes* Mech Dev 2004 121 605 618 10.1016/j.mod.2004.03.012 15210170

[B24] Swarup H Stages in the Development of the Stickleback *Gasterosteus aculeatus* (L.) J Embryol Exp Morphol 1958 6 373 383 13575650

[B25] Newport J Kirschner M A major developmental transition in early *Xenopus* embryos Cell 1982 30 675 686 10.1016/0092-8674(82)90272-0 6183003

[B26] Gilbert SF Developmental Biology 2000 Sunderland (MA): Sinauer Associates

[B27] Graham CF Morgna RW Changes in the cell cycle during early amphibian development Developmental Biology 1966 14 439 460 10.1016/0012-1606(66)90024-8

[B28] Sakaue-Sawano A Kurokawa H Morimura T Hanyu A Hama H Osawa H Kashiwagi S Fukami K Miyata T Miyoshi H Imamura T Ogawa M Masai H Miyawaki A Visualizing spatiotemporal dynamics of multicellular cell-cycle progression Cell 2008 132 487 498 10.1016/j.cell.2007.12.033 18267078

[B29] Sugiyama M Sakaue-Sawano A Iimura T Fukami K Kitaguchi T Kawakami K Okamoto H Higashijima S Miyawaki A Illuminating cell-cycle progression in the developing zebrafish embryo Proc Natl Acad Sci USA 2009 106 20812 20817 10.1073/pnas.0906464106 19923430 PMC2779202

[B30] Kane DA Kimmel CB The zebrafish midblastula transition Development 1993 119 447 456 8287796 10.1242/dev.119.2.447

[B31] Kraeussling M Wagner TU Schartl M Highly asynchronous and asymmetric cleavage divisions accompany early transcriptional activity in pre-blastula medaka embryos PLoS One 2011 6 e21741 10.1371/journal.pone.0021741 21750728 PMC3131289

[B32] Mathavan S Lee SG Mak A Miller LD Murthy KR Govindarajan KR Tong Y Wu YL Lam SH Yang H Ruan Y Korzh V Gong Z Liu ET Lufkin T Transcriptome analysis of zebrafish embryogenesis using microarrays PLoS Genet 2005 1 260 276 16132083 10.1371/journal.pgen.0010029 PMC1193535

[B33] Podrabsky JE Garrett ID Kohl ZF Alternative developmental pathways associated with diapause regulated by temperature and maternal influences in embryos of the annual killifish *Austrofundulus limnaeus* J Exp Biol 2010 213 3280 3288 10.1242/jeb.045906 20833920 PMC2936969

[B34] Polačik M Blažek R Režucha R Vrtílek M Terzibasi Tozzini E Reichard MJ Alternative intrapopulation life-history strategies and their trade-offs in an African annual fish Evol Biol 2014 27 854 865 10.1111/jeb.12359 24666645

